# Association Between State Regulations Supportive of Third-party Services and Likelihood of Assisted Living Residents in the US Dying in Place

**DOI:** 10.1001/jamahealthforum.2022.3432

**Published:** 2022-10-07

**Authors:** Emmanuelle Belanger, Nicole Rosendaal, Xiao (Joyce) Wang, Joan M. Teno, David M. Dosa, Pedro L. Gozalo, Paula Carder, Kali S. Thomas

**Affiliations:** 1 Department of Health Services, Policy & Practice, Brown University School of Public Health, Providence, Rhode Island; 2 Center for Gerontology and Healthcare Research, Brown University School of Public Health, Providence, Rhode Island; 3 Division of General Internal Medicine and Geriatrics, Oregon Health & Science University, Portland; 4 US Department of Veterans Affairs Medical Center, Providence, Rhode Island; 5 The Warren Alpert Medical School of Brown University, Providence, Rhode Island; 6 Institute on Aging, School of Public Health, Oregon Health and Science University–Portland State University, Portland

## Abstract

**Importance:**

Older adults are increasingly residing in assisted living residences during their last year of life. The regulations guiding these residential care settings differ between and within the states in the US, resulting in diverse policies that may support residents who wish to die in place.

**Objective:**

To examine the association between state regulations and the likelihood of assisted living residents dying in place. The study hypothesis was that regulations supporting third-party services, such as hospice, increase the likelihood of assisted living residents dying in place.

**Design, Setting, and Participants:**

This retrospective cohort study combined data about assisted living residences in the US from state registries with an inventory of state regulations and administrative claims data. The study participants comprised 168 526 decedents who were Medicare beneficiaries, resided in 8315 large, assisted living residences (with ≥25 beds) across 301 hospital referral regions during the last 12 months of their lives, and died between 2017 and 2019. Descriptive analyses were performed at the state level, and 3-level multilevel models were estimated to examine the association between supportive third-party regulations and dying in place in assisted living residences. The data were analyzed from September 2021 to August 2022.

**Exposures:**

Supportive (vs “silent,” ie, not explicitly mentioned in regulatory texts) state regulations regarding hospice care, private care aides, and home health services, as applicable to licensed/registered assisted living residences across the US.

**Main Outcomes and Measures:**

Presence in assisted living residences on the date of death.

**Results:**

The median (IQR) age of the 168 526 decedents included in the study was 90 (84-94) years. Of these, 110 143 (65.4%) were female and 158 491 (94.0%) were non-Hispanic White. Substantial variation in the percentage of assisted living residents dying in place was evident across states, from 18.0% (New York) to 73.7% (Utah). Supportive hospice and home health regulations were associated with a higher odds of residents dying in place (adjusted odds ratio [AOR], 1.38; 95% CI, 1.24-1.54; *P* < .001; and AOR, 1.21; 95% CI, 1.10-1.34; *P* < .001, respectively). In addition, hospice regulations remained significant in fully adjusted models (AOR, 1.46; 95% CI, 1.25-1.71).

**Conclusions and Relevance:**

The findings of this cohort study suggest that a higher percentage of assisted living residents died in place in US states with regulations supportive of third-party services. In addition, assisted living residents in licensed settings with regulations supportive of hospice regulations were especially likely to die in place.

## Key Points

**Question:**

Are state regulations supportive of third-party services associated with the likelihood of assisted living residents dying in place?

**Findings:**

In this cohort study of 168 526 decedents who received care in 8315 assisted living residences in the US, the individuals who resided in residences operating under a license with supportive hospice regulations were found to be 1.46 times more likely to die in place.

**Meaning:**

The findings of this study suggest that state regulations supportive of third-party services in assisted living residences may be associated with the likelihood of residents dying in place.

## Teaser

This cohort study examines the association between state regulations supportive of third-party services and the likelihood of assisted living residents in the US dying in place.

## Introduction

An increasing number of older adults in the US reside in assisted living, a residential care setting focusing on supportive care rather than nursing care, during their last year of life. The exact number of residents remaining in assisted living until death has been difficult to ascertain nationally because of a lack of federally mandated assessments for residents and because such residences are not recorded as a specific place of death in data from the National Center for Health Statistics.^1,2^ By 2015, residents who were Medicare beneficiaries and were receiving hospice services in assisted living at the time of death represented 18% of the beneficiaries dying in community settings.^3^ A novel method to identify the Medicare beneficiaries residing at validated zip codes for large assisted living residences has contributed to better documentation of national trends^4^ and uncovered substantial national variation in the end-of-life care trajectories of assisted living decedents across states in 2016, ranging from Utah, where decedents spent an average of 24.0 days in assisted living during the last month of life, to North Dakota with 13.8 days.^4,5^

State regulations governing the operation of licensed or registered assisted living residences vary across the US.^6,7^ These regulations pertain to various aspects of the care provided, from admission criteria to staffing requirements. They also allow and/or restrict third-party services such as hospice, home health services, and private care aides, which may play an important role in meeting the growing care needs of persons who wish to die in place. Ball et al^8^ explored end-of-life care provided in 8 assisted living residences in Atlanta, Georgia, and concluded that access to hospice services improved the ability of assisted living staff to meet the needs of residents with deteriorating health conditions without the need to transfer them to an acute care facility, a nursing home, or an inpatient hospice facility. Previous work by Belanger et al^9^ using a national cohort of assisted living decedents confirmed a significant association between regulations supportive of hospice care in the assisted living setting and residents’ hospice utilization during the last month of life. Similarly, a recent study based in Oregon comparing the supportive care needs of more than 1100 assisted living residents with and without hospice services during the 90 days prior to the survey measure concluded that hospice recipients were on average older and more likely to need help with activities of daily living and require care overnight.^10^ These findings confirm that third-party services, such as hospice care, may likely supplement the care provided in assisted living whenever residents’ health conditions deteriorate.

In an aging population, assisted living increasingly becomes “home” for frail, older individuals in the US who prefer dying at home. However, there is a paucity of research about the association between state regulations and the likelihood of assisted living residents to die in place.

This study has 2 objectives: (1) to describe the national variation at the state level in the regulation of third-party services and the percentage of assisted living residents dying in place, and (2) to examine the extent to which these regulations are associated with the likelihood of residents dying in place. We hypothesized that any regulations supportive of third-party services, such as hospice care, may be associated with a higher likelihood that a given resident remains in place until death, and that hospice may likely be the most important regulation regarding dying in place, relative to other third-party services (ie, home health or private care aides).

## Methods

This study combined publicly available data from state assisted living registries with an in-depth content analysis of assisted living state regulations and administrative claims data from Medicare beneficiaries who died between 2017 and 2019 and resided in assisted living residences with 25 beds or more at some point during their last 12 months of life. We mapped assisted living regulations pertaining to third-party services, specifically hospice, home health services, and private care aides, and compared these regulations with geographic variations in the outcome of interest. We also estimated multilevel logistic regression models to examine the association between residing in an assisted living residence operating under these regulations and the likelihood of dying in place, accounting for nested observations of residents in assisted living residences with shared regulations, in hospital referral regions (HRRs). This study used public use files and secondary administrative claims data and was therefore exempt from review according to the institutional review board (IRB) at Brown University, Providence, Rhode Island. This IRB also waived the need for informed consent because the data were deidentified secondary administrative claims.

### Data Sources and Study Population

We retrieved information about assisted living residences in the US, including license type, unique 9-digit zip codes, and capacity, from publicly available 2017 state licensing registries.^4^ Registries were combined with a thorough inventory of assisted living regulations about third-party services in effect in 2018 by license type.^7^ Considering the relatively small number of residents dying at each assisted living residence annually, the decedents were pooled across 3 study years from 2017 to 2019. The decedent cohort was developed using the annual Master Beneficiary Summary Files to identify beneficiaries who died during the study period and resided at a previously validated assisted living zip codes during their last 12 months of life. The Master Beneficiary Summary Files contain race and ethnicity as documented by the Social Security Administration. Although all decedents were retained in state-level descriptions, the multilevel models were restricted to assisted living residences with at least 5 decedents during the study period to ensure stability of multilevel estimates. Connecticut and Minnesota were also excluded from these analyses because of a different licensing structure preventing the match of residents to specific assisted living residences.^11^

### Study Measures

We examined a binary outcome variable of whether a given assisted living resident died in place. To identify the place of death, we used the Resident History File,^12^ an algorithm based on extensive administrative claims data developed at Brown University to track daily health care utilization and residential setting before death. In order of certainty, we checked for inpatient hospitalization as indicated in MedPAR files, skilled nursing facility claims and/or presence of a minimum data set assessment, or general inpatient hospice or respite care as indicated in hospice claims. In the absence of these claims or assessments, residents were considered to have remained in assisted living on each given day. Any resident with hospice who was transferred out of assisted living to general inpatient hospice during the last 7 days of life was also considered as having died in place. This is because general inpatient hospice, as a higher level of hospice care for symptom control, cannot be provided in assisted living residences and represents a hospice’s decision to transfer the resident based on care needs. We also conducted a sensitivity analysis considering transfer to inpatient hospice in the last 7 days of life as not dying in place to assess if this decision altered the association of interest.

For this study, we examined 3 regulations supportive of third-party services delivery from the regulation inventory as potentially relevant to increasing health care services for residents approaching the end of life: (1) regulations allowing residents to use hospice care in assisted living and/or to be admitted to assisted living while already on hospice; (2) regulations allowing residents to use home health services; and (3) regulations allowing residents to hire private care aides. Use of private care aides is notably absent from administrative claims data sources. Previous work^7^ has shown that the structure of assisted living licensing is complex and that different types of licenses, such as primary licenses or designations, have different sets of regulations, some of which apply to the entire assisted living residence whereas others affect specific subsets of residents. For these analyses, we considered that if any active license or designation at a given assisted living residence was supportive of third-party services, all residents at this location were potentially affected by these regulations.

### Statistical Analyses

The data were analyzed from September 2021 to August 2022. Relevant assisted living characteristics, as well as the distribution of regulations of interest were explored with descriptive statistics. We characterized state-level patterns of the regulations of interest (supportive, inconsistent across licenses within a state, and “silent,” ie, not explicitly mentioned in regulatory texts), as well as the distribution of our outcome variable by state. We then estimated 3-level multilevel models of decedents nested within assisted living residences, which were themselves nested within HRRs to account for shared assisted living residence and market-level factors. We tested each of the independent regulation variables to examine the likelihood of dying in place, unadjusted and then adjusted for the resident’s demographic characteristics (age, sex, and race and ethnicity). We also estimated adjusted and unadjusted multilevel models with all regulations in a single model, to examine whether any of these third-party services regulations were a more important factor for dying in place. We conducted a sensitivity analysis that considered those individuals who were transferred in the last 7 days of life from hospice in assisted living to an inpatient hospice as not having died in place (eTable 1 in the Supplement). Data were analyzed using Stata, version 17 (StataCorp LLC).

## Results

### State-Level Patterns

Table 1 provides descriptive characteristics of the full cohort of decedents (N = 175 414), and the analytical cohort in assisted living residences with 5 or more decedents during the study period (n = 168 526). Of those in the analytical cohort, the median (IQR) age was 90 (84-94) years, 110 143 (65.4%) were female, 58 383 (34.6%) were male, and 158 491 (94.0%) were non-Hispanic White.

**Table 1.** Descriptive Characteristics of Medicare Decedents in Large Assisted Living Residences in the US in the Last 12 Months of Life, 2017 to 2019

Characteristic	National sample included in state-level results (N = 175 414)	Analytical sample included in multilevel models (n = 168 526)^a^
Characteristics		
Age, median (IQR), y	89 (84-94)	90 (84-94)
Sex, No. (%)		
Male	60 915 (34.7)	58 383 (34.6)
Female	114 499 (65.3)	110 143 (65.4)
Race and ethnicity, No. (%)		
African American	6398 (3.6)	6096 (3.6)
Hispanic	1165 (0.7)	1111 (0.7)
Non-Hispanic White	164 880 (94.0)	158 491 (94.0)
Other or unknown^b^	2971 (1.7)	2828 (1.7)
Outcome, No. (%)		
Died in assisted living	76 604 (43.7)	73 021 (43.3)
Resided in an assisted living residence with regulations supportive of		
Services, No. (%)		
Hospice	145 094 (82.7)	139 748 (82.9)
Home health	134 586 (76.7)	129 578 (76.9)
Private care aides, No. (%)	58 615 (33.4)	56 429 (33.5)

^a^ Assisted living residences with at least 5 decedents.

^b^ Other included Asian, North American Native, and other.

**Figure 1.**
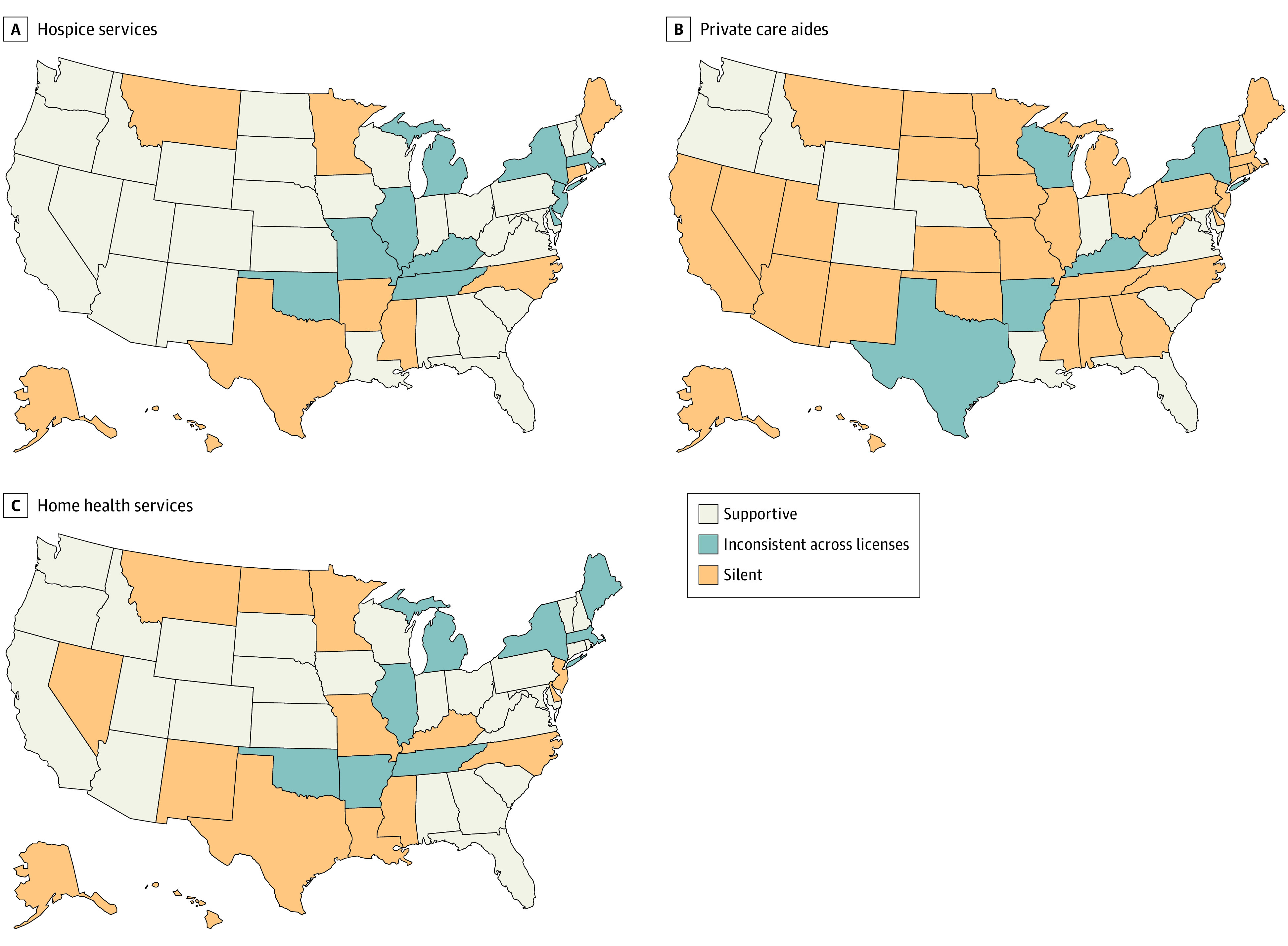
Characteristics of State Regulations About Third-party Services Pertaining to Assisted Living Residences Policies for Connecticut and Minnesota are presented in this map, but these states were not otherwise included in the analyses.

**Figure 2.**
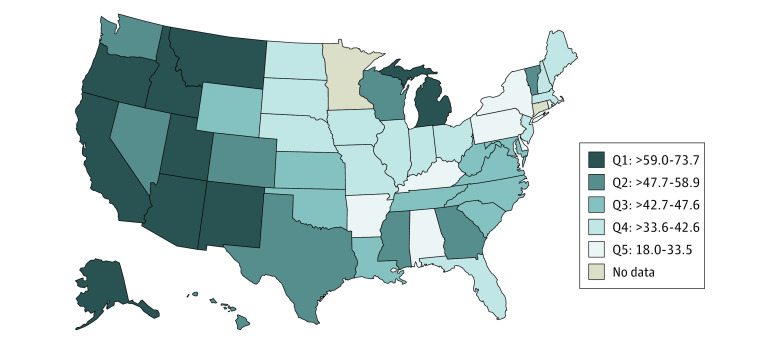
Statewise Percentage of Assisted Living Decedents Dying in Place Q1 through Q5 indicate quintiles 1 through 5.

The distribution of regulations varied across the US (Figure 1), with 13 states supporting all third-party services across all licenses (eg, Florida, New Hampshire, Oregon, and Washington). Many states were either supportive of only some third-party services (eg, California) or their regulations varied between license types or classifications (eg, New York). In addition, 6 states (Alaska, Hawaii, Minnesota, Mississippi, Montana, and North Carolina), remained silent on all third-party regulations across assisted living licenses in effect. Figure 2 shows the percentage of assisted living residents dying in place and suggests that residences with regulations supportive of third-party services tend to retain a larger share of residents until death. A substantial variation was observed in the percentage of assisted living residents dying in place across states, from 18.0% (New York) to 73.7% (Utah). Notable exceptions to these findings included Alaska and Montana, which were fully silent about third-party services regulations and yet ranked high in terms of residents dying in place. Other exceptions included South Dakota, Nebraska, Rhode Island, Pennsylvania, Ohio, and Indiana, all of which had supportive third-party regulations but ranked in the lower quintiles of the outcome. Additional state-level data and the percentage of decedents residing in settings with supportive vs silent regulations in states that have inconsistent regulations across license types are provided in eTable 1 in the Supplement.

### Multilevel Sample Descriptions

Table 1 provides the distribution of the independent variables for our analytical sample of 168 526 individuals who were Medicare beneficiaries, died between 2017 and 2019, and were cared for by 8315 assisted living residences during the last 12 months of their life. Although only 3.9% of decedents nationally were excluded from the multilevel analyses because of the requirement of a minimum of 5 decedents per residence, this led to the exclusion of 29.7% of assisted living residences, which shows the uneven distribution of decedents across residential care settings. The residences retained in the analytical sample had a combination of 146 primary, sublicense, or designation license classifications with relevant regulatory information about third-party services.

 Among the fee-for-service beneficiaries (69.9% of the total analytical sample), 72.3% had a diagnosis of Alzheimer disease and related dementias. Among the decedents, 82.9% resided in assisted living residences with regulations supportive of hospice, 76.9% resided in residences with supportive home health regulations, and 33.5% resided in residences that allowed private care aides. In terms of the outcome variable, 43.3% of the decedents died in assisted living residences. Medicare beneficiaries were more likely to die in place when residing in assisted living residences with regulations supportive of hospice services (45.0% vs 36.3% for silent hospice regulations), and home health services (45.0% vs 37.9% for silent home health regulations).

### Association of Third-party Regulations and Medicare Beneficiaries Dying in Assisted Living

Medicare beneficiaries residing in licensed settings with regulations supportive of hospice and home health services were more likely to die in place (adjusted odds ratios [AORs], 1.38; 95% CI, 1.24-1.54; *P* < .001; and 1.21; 95% CI, 1.10-1.34; *P* < .001, respectively) (Table 2). Regulations allowing residents to hire private care aides were not significantly associated with a higher likelihood of dying in place (AOR, 1.08; 95% CI, 0.98-1.18; *P* = .13). As given in Table 2, intraclass correlations were substantial at both the assisted living residence (0.24), and the HRR (0.09), confirming the importance of controlling for nested observations. Only supportive hospice regulations remained significant when a single adjusted model with all 3 regulations was estimated (OR, 1.46; 95% CI, 1.25-1.71; *P* < .001), supporting our hypothesis that hospice may be the most important third-party service for residents to die in place. The correlation between regulations supportive of hospice and home health regulations was 0.77 (95% CI, 0.77-0.77) in our data, compared with 0.26 (95% CI, 0.26-0.27) between hospice and private care aides and 0.29 (95% CI, 0.28-0.29) between home health and private care aides. The sensitivity analysis classifying residents as not having died in place if they transferred to a hospice inpatient facility in the last week of life yielded similar results (eTable 2 in the Supplement).

**Table 2.** Odds Ratios of Assisted Living Residents Dying in Place From 3-Level Multilevel Regression Model

	Unadjusted (n = 168 526)				Adjusted (n = 168 526)^a^			
	OR (95% CI)	*P* value	Residual intraclass correlation (95% CI)		OR (95% CI)	*P* value	Residual intraclass correlation (95% CI)	
			AL level	HRR level			AL level	HRR level
Regulations supportive of								
Hospice services	1.44 (1.30-1.61)	<.001	0.25 (0.23-0.26)	0.09 (0.08-0.11)	1.38 (1.24-1.54)	<.001	0.24 (0.23-0.26)	0.09 (0.08-0.11)
Home health services	1.25 (1.13-1.38)	<.001	0.25 (0.24-0.27)	0.09 (0.08-0.11)	1.21 (1.10-1.34)	<.001	0.25 (0.23-0.26)	0.09 (0.08-0.11)
Private care aides	1.10 (1.00-1.21)	.06	0.25 (0.24-0.27)	0.10 (0.08-0.11)	1.08 (0.98-1.18)	.13	0.25 (0.23-0.26)	0.10 (0.08-0.12)
Multivariable model—all regulations in 1 model								
Hospice services	1.52 (1.30-1.78)	<.001	0.25 (0.23-0.26)	0.09 (0.08-0.11)	1.46 (1.25-1.71)	<.001	0.24 (0.23-0.26)	0.09 (0.08-0.11)
Home health services	0.94 (0.81-1.09)	.42			0.94 (0.81-1.09)	.42		
Private care aides	0.98 (0.89-1.09)	.71			0.98 (0.88-1.08)	.63		

Abbreviations: AL, assisted living; HRR, hospital referral region; OR, odds ratio.

^a^ Adjusted for age, sex, and race and ethnicity.

## Discussion

With an increasing number of older adults calling assisted living “home” and wishing to remain in place at the end of life, there is an increasing concern about the level of oversight needed to protect a vulnerable population of terminally ill residents. This study examined the association between state regulations for third-party services in assisted living residences, particularly hospice, home health, and private care aides, and the likelihood of assisted living residents dying in place. The findings support our hypothesis that assisted living residents in settings operating under licenses that are supportive of third-party services may, on average, be more likely to die in place. Supportive hospice regulations were found to be most important to dying in place. These results remained significant in a multilevel model accounting for shared factors at the levels of assisted living residence and HRR, such as assisted living internal protocols and/or strong hospice markets. Our findings are also consistent with results from the Medicare Current Beneficiary Survey^13^ showing that, between 2002 and 2018, a higher proportion of residents died in place in community-based residential settings that allowed clinical services.

The results of the present study revealed expected state-level patterns in terms of the likelihood of residents dying in place, with a larger proportion of individuals remaining in assisted living on the date of death in states with supportive third-party policies across license types. However, an “average state” does not exist, and there were notable outliers with supportive policies but low retention, and with a lack of supportive policies but high retention. Regulatory “silence” can be intentional,^14^ because silence does not necessarily prohibit licensees’ actions, such as third-party use, but could make assisted living residences more likely to encourage residents to relocate when their health deteriorates. The observed trends may also likely reflect the underlying geographic variation in sociocultural attitudes toward end-of-life care. Prior research has documented the many challenges of meeting the increasing care needs of terminally ill assisted living residents in specific states^15,16,17^; however, extensive national variation in the end-of-life care outcomes of assisted living residents remains to be further explained.^5^ There is also an increasing knowledge about the extent of variation in state regulations for assisted living^6^ and their enforcement.^18^ It is possible that third-party services interact with other relevant policies across states, such as health care providers’ scope of practice or Medicaid state plans (eg, North Carolina), to assess the likelihood of remaining in place toward the end of life with the support of additional services and financial support to pay for that care.

Of note, despite evidence that older adults may prefer to avoid nursing home admission close to death,^19^ presence in assisted living on the day of death is not a guarantee of high-quality end-of-life care. There is also general agreement about the difficulty of predicting end-of-life disease trajectories among frail older adults, and especially the individuals with dementia, making the management of care transitions to and from assisted living particularly challenging near the end of life. For instance, researchers from the United Kingdom followed 121 residents of 6 residential care homes and examined the end-of-life care trajectories of 23 residents who died during their study period. They concluded that, although some residents were recognized as approaching the end of life, more than half either died unexpectedly or after considerable prognostic uncertainty following one or more acute hospitalizations.^20^ We recently reported that slightly more than 1 in 10 assisted living decedents in 2018 died in place without receiving hospice services.^9^ Moreover, lower bereaved next-of-kin quality ratings have raised concerns about the quality of hospice care provided in residential care settings.^21^ The retention of dying assisted living residents and the quality of care provided to them at the end of life deserve additional research and are likely the result of a complex interplay between state regulations, long-term care, and hospice markets as well as the needs, preferences, and resources of residents and their families.

### Strengths and Limitations

This study has a few limitations. This cross-sectional study design does not allow us to ascertain whether supportive regulations came first or whether assisted living residences whose residents prefer to die in place have indicated the need for more supportive third-party policies. The generalizability of our results is limited to assisted living residences with a capacity of 25 beds or more, and those with at least 5 decedents between 2017 and 2019. As of 2016, the National Center for Health Statistics estimated that large, assisted living residences represented 84.1% of all licensed beds, and one would expect smaller residences to have low numbers of decedents each year.^22^ The estimates reported were also not adjusted for any factors beyond regulations and demographic characteristics of residents, given that important covariates are not readily available across state registries nationally. Several residence-level characteristics, such as staff knowledge about dementia,^23^ and resident-level clinical factors, such as the availability of family caregivers,^24^ are associated with end-of-life transitions among dying assisted living residents, and these aspects should be explored further. Important social determinants of health are not available in administrative claims data, and chronic conditions are poorly documented among Medicare beneficiaries enrolled in managed care plans. Finally, we examined assisted living regulations in only 2018 because of data availability, but do not expect these regulations to have changed significantly during the study period based on our assessment of the previous 5 years.

Despite these limitations, our study has several strengths. It includes a national cohort to provide an overall perspective of the association between extensive state regulations of assisted living residences and the likelihood of residents dying in place.

## Conclusions

In this cohort study, we observed that state regulations supportive of third-party services were associated with a higher likelihood of assisted living residents dying in place, both in descriptive state-level trends and in adjusted multilevel models. Further research is needed to identify the determinants of place of death and quality of end-of-life care for assisted living residents, but supportive regulations for services that are important to meet the increasing care needs of individuals approaching death may be a good place to start among the many states where there is still regulatory uncertainty about these services.

## Notes

Supplement. **eTable 1.** Percentage of Residents Dying in Place and Residing in Licensed Settings With Specific Regulations by State **eTable 2.** Odds Ratios of Assisted Living Residents Dying in Place From 3-Level Multilevel Regression Model (Decedents Transferring to Inpatient Hospice in the Last 7 Days of Life Not Counted as Dying in Place)
