# Supplementary material for: Association Between State Regulations Supportive of Third-party Services and Likelihood of Assisted Living Residents in the US Dying in Place
Source: JAMA Health Forum. 2022 Oct 7;3(10):e223432. doi: 10.1001/jamahealthforum.2022.3432 (PMC9547316; doi:10.1001/jamahealthforum.2022.3432)
Supplement: Supplement. — eTable 1. Percentage of Residents Dying in Place and Residing in Licensed Settings With Specific Regulations by State eTable 2. Odds Ratios of Assisted Living Residents Dying in Place From 3-Level Multilevel Regression Model (Decedents Transferring to Inpatient Hospice in the Last 7 Days of Life Not Counted as Dying in Place) [file jamahealthforum-e223432-s001.pdf]

Supplementary Online Content

Belanger E, Rosendaal N, Wang X, et al. Association between state regulations supportive of third-party services and likelihood of assisted living residents in the US dying in place. *JAMA Health Forum.* 2022;3(10):e223432. doi:10.1001/jamahealthforum.2022.3432

eTable 1. Percentage of Residents Dying in Place and Residing in Licensed Settings with Specific Regulations by State

eTable 2. Odds Ratios of Assisted Living Residents Dying in Place from 3-Level Multilevel Regression Model (Decedents Transferring to Inpatient Hospice in the Last 7 Days of Life Not Counted as Dying in Place)

This supplementary material has been provided by the authors to give readers additional information about their work.

eTable 1. Percentage of Residents Dying in Place and Residing in Licensed Settings with Specific Regulations by State

State	Percentage of residents dying present in assisted living on the date of death	Hospice regulations	Home health regulations	Private care aides regulations
**Alaska**	62.7%	silent	silent	silent
**Alabama**	33.1%	supportive	supportive	silent
**Arkansas**	29.6%	silent	34.7% supportive [a]	19.7% supportive
**Arizona**	64.4%	supportive	supportive	silent
**California**	59.1%	supportive	supportive	silent
**Colorado**	48.0%	supportive	supportive	supportive
**Connecticut**	No data	silent	supportive	silent
**DC**	49.0%	supportive	supportive	supportive
**Delaware**	33.5%	90.2% supportive	silent	silent
**Florida**	40.5%	supportive	supportive	supportive
**Georgia**	54.3%	supportive	supportive	silent
**Hawaii**	47.6%	silent	silent	silent
**Iowa**	30.5%	supportive	supportive	silent
**Idaho**	65.0%	supportive	supportive	supportive
**Illinois**	42.6%	86.1% supportive	86.1% supportive	silent
**Indiana**	41.2%	supportive	supportive	supportive
**Kansas**	46.1%	supportive	supportive	silent
**Kentucky**	32.1%	66.2% supportive	silent	66.2% supportive
**Louisiana**	46.5%	supportive	silent	supportive
**Massachusetts**	35.6%	90.7% supportive	90.7% supportive	silent
**Maryland**	42.7%	supportive	supportive	supportive
**Maine**	36.1%	silent	70.6% supportive	silent
**Michigan**	59.4%	99.7% supportive	99.7% supportive	silent
**Minnesota**	No data	silent	silent	silent
**Missouri**	35.1%	12.7% supportive	silent	silent
**Mississippi**	49.2%	silent	silent	silent
**Montana**	61.4%	silent	silent	silent
**North Carolina**	46.8%	silent	silent	silent
**North Dakota**	18.6%	supportive	silent	silent
**Nebraska**	38.8%	supportive	supportive	supportive
**New Hampshire**	41.8%	supportive	supportive	supportive
**New Jersey**	38.6%	95.8% supportive	silent	silent
**New Mexico**	60.2%	supportive	silent	silent

**Nevada**	54.7%	supportive	silent	silent
**New York**	18.0%	33.7% supportive	33.7% supportive	33.7% supportive
**Ohio**	34.0%	supportive	supportive	silent
**Oklahoma**	46.6%	92.7% supportive	92.7% supportive	silent
**Oregon**	70.8%	supportive	supportive	supportive
**Pennsylvania**	31.7%	supportive	supportive	silent
**Rhode Island**	25.3%	supportive	supportive	silent
**South Carolina**	46.1%	supportive	supportive	supportive
**South Dakota**	27.2%	supportive	supportive	silent
**Tennessee**	44.9%	96.3% supportive	3.8% supportive	silent
**Texas**	48.4%	silent	silent	18.4% supportive
**Utah**	73.7%	supportive	supportive	silent
**Virginia**	47.6%	supportive	supportive	supportive
**Vermont**	58.9%	supportive	supportive	silent
**Washington**	55.1%	supportive	supportive	supportive
**Wisconsin**	53.6%	supportive	supportive	59.7% supportive
**West Virginia**	45.6%	supportive	supportive	silent
**Wyoming**	45.2%	supportive	supportive	supportive

[a]For inconsistent states in which some licenses are supportive and some are silent on regulations, the percentage of decedents in this state that lived in ALs with supportive regulations is provided in this table.

eTable 2. Odds Ratios of Assisted Living Residents Dying in Place from 3-Level Multilevel Regression Model (Decedents Transferring to Inpatient Hospice in the Last 7 Days of Life Not Counted as Dying in Place)

	**Unadjusted (N=168,526)**			**Adjusted[a] (N=168,526)**		
	Odds Ratios (95% CI)	*P* value	Residual intraclass correlation (95% CI)	Odds Ratios (95% CI)	*P* value	Residual intraclass correlation (95% CI)
Regulations Supportive of:						
**Hospice services**	1.43 (1.29-1.60)	<0.001		1.37 (1.24-1.53)	<0.001	
			AL level: 0.25 (0.24-0.27)			*AL level: 0.25 (0.23-0.26)*
			HRR level: 0.10 (0.08-0.11)			*HRR level: 0.10 (0.08-0.12)*
**Home health services**	1.25 (1.13-1.38)	<0.001		1.21 (1.10-1.34)	<0.001	
			AL level: 0.25 (0.24-0.27)			*AL level: 0.25 (0.23-0.26)*
			HRR level: 0.10 (0.08-0.12)			*HRR level: 0.10 (0.08-0.12)*
**Private care aides**	1.08 (0.98-1.19)	0.136		1.06 (0.96-1.16)	0.259	
			AL level: 0.25 (0.24-0.27)			*AL level: 0.25 (0.23-0.26)*
			HRR level: 0.10 (0.08-0.12)			*HRR level: 0.10 (0.08-0.12)*
Multivariable model - All Regulations in One Model						
**Hospice services**	1.52 (1.29-1.78)	<0.001		1.45 (1.24-1.70)	<0.001	
**Home health services**	0.95 (0.82-1.10)	0.487		0.95 (0.82-1.10)	0.486	
**Private care aides**	0.96 (0.87-1.07)	0.465		0.96 (0.86-1.06)	0.394	
			AL level: 0.25 (0.24-0.27)			*AL level: 0.25 (0.23-0.26)*
			HRR level: 0.10 (0.08-0.12)			*HRR level: 0.10 (0.08-0.12)*

[a]Adjusted for age, sex, and race/ethnicity.

Abbreviations: CI, Confidence intervals; AL, assisted living; HRR, hospital referral region.
